# Inverse-designed gyrotropic scatterers for non-reciprocal analog computing

**DOI:** 10.1515/nanoph-2025-0247

**Published:** 2025-11-28

**Authors:** Nikolas Hadjiantoni, Heedong Goh, Stephen M. Hanham, Miguel Navarro-Cía, Andrea Alù

**Affiliations:** School of Engineering, 1724 University of Birmingham, Birmingham, UK; Seoul National University, Seoul, South Korea; Photonics Initiative, Advanced Science Research Center, City University of New York, New York, USA; Department of Materials, Imperial College London, London, UK; School of Physics and Astronomy & School of Engineering, University of Birmingham, Birmingham, UK; Physics Program, Graduate Center & Photonics Initiative, Advanced Science Research Center, City University of New York, New York, USA

**Keywords:** analog computer, inverse design, non-reciprocity

## Abstract

While conventional von Neumann based machines are increasingly challenged by modern day requirements, electromagnetic analog computing devices promise to provide a platform that is highly parallel, efficient and fast. Along this paradigm, it has been shown that arrays of subwavelength electromagnetic scatterers can be used as solvers of partial differential equations. Inverse design offers a powerful tool to synthesize such analog computing machines, utilizing engineered non-local responses to produce the solution of a desired mathematical operation encoded in the scattered fields. So far, this approach has been largely restricted to linear, reciprocal scatterers, limiting its generality and applicability. Here we demonstrate how arrays of gyrotropic scatterers can be used to solve a more general class of differential equations. Through inverse design, with a combination of evolutionary and gradient based algorithms, the position of the scatterers is optimized to achieve the desired kernel response. Introducing gyrotropic media, we also demonstrate improved accuracy by >2 orders of magnitude compared to similarly sized reciprocal systems designed with the same method.

## 1 Introduction

While the demand to process data is increasing at an unprecedented rate, von Neumann based computers are proving to be simultaneously energy inefficient and overly time consuming [1], [2]. Between 2011 and 2022, the average computation required by machine learning systems has increased from 10^16^ floating point operations per second (FLOPS) to 10^24^ FLOPS, [3]. This indicates a significant need to increase the energy efficiency and time cost of mathematical operations.

In an attempt to remove such a bottleneck from the development of computational models, recent works have explored the use of photonic scatterers, metamaterials and metasurfaces to take advantage of their fast and highly efficient response to model integro-differential problems, recurrent neural networks (RNNs) and extreme learning machines [2], [4], [5], [6], [7], [8], [9], [10], [11], [12], [13], [14]. Since such optical systems yield massively parallel, high-speed and energy efficient operations, these photonic devices have proven to be a promising surrogate for their von Neumann based counterparts [1].

**Figure 1:**
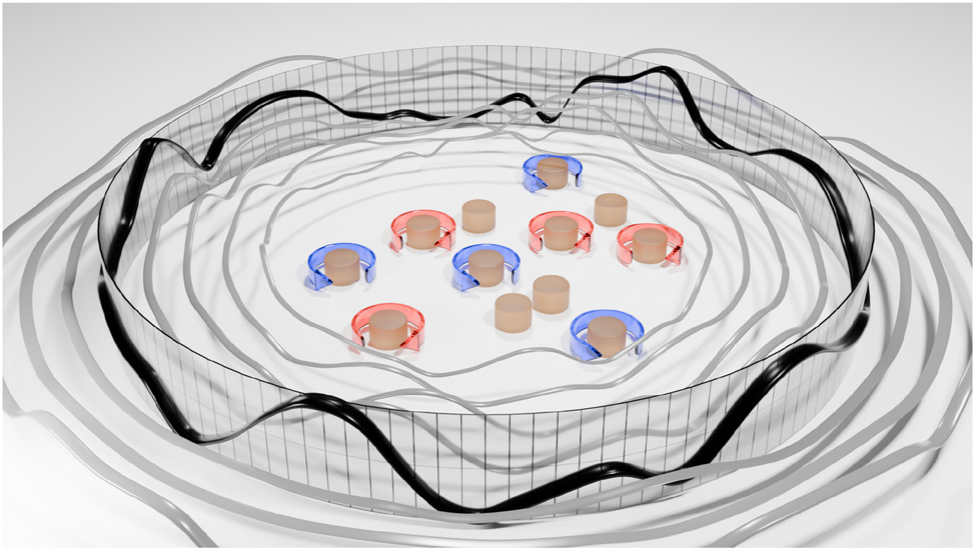
Non-reciprocal scatterer composed of two types of cylinders, gyrotropic and dielectric cylinders, returning a solution *u* = *A* ^−1^ *f* encoded within a scattered wave.

By embedding the underlying mathematical problem in the properties of the incoming field, analog computing devices can shape the field distribution by fine-tuning their spatial and temporal parameters to achieve a desired response [12]. The response can be thought of as a kernel acting on the input distribution that approximates a linear mathematical operator or its inverse. In this context, inverse design has been drawing significant interest in the quest of developing analog computing optical machines, due to its ability to find non-trivial solutions for such scatterers [5], [15], [16], [17]. As shown in previous work [8], odd-symmetric mathematical operators require breaking both transverse and longitudinal symmetries. For this reason, inverse-designed structures with broken symmetries offer an ideal design playground for these devices [18]. So far, however, they have been limited to the solution of linear integro-differential equations [19].

As another limitation, the meta-structures considered so far have relied on reciprocal media, where reciprocity indicates the inherent symmetry in optical response as source and observation points are exchanged. While a good number of relevant mathematical problems obey sufficient symmetries to be synthesized using reciprocal optical responses, breaking reciprocity can enable a wider range of operations, relevant for instance in the context of machine learning, thermodynamics and cosmology [20], [21], [22]. In this manuscript, we introduce an analog computing framework that harnesses nonreciprocal scattering induced by a static magnetic bias on an array of scatterers (Figure 1), with the goal of expanding the rational synthesis of analog optical computers based on engineered nonlocalities.

## 2 Methods

Here, we describe the analytical procedures for formulating the wave–scatterer interaction into a scattering-matrix representation and for discretizing the target mathematical problem into a matrix form. Subsequently, we establish the relationship between the two system matrices, which guides the design process of the scatterer.

### 2.1 Ensemble of gyrotropic particles

The geometry for the proposed analog computing framework is described by a two-dimensional problem in which we assume that the fields are transverse electric (TE), which can be reduced to a scalar Helmholtz equation in terms of the magnetic field component *H*_*z*_. We compose the scatterer by a system of small particles, where each particle is approximated by a dipole moment.

The polarizability of each particle is derived from the dominant scattering coefficients. We consider a single circular scatterer in a vacuum with radius *a*. The solution to an incident plane wave with *e* ^*iωt*^ dependency reads

(1) $$\begin{array}{cc} H_{z}\left(r,\phi \right) & =H_{z}^{inc}\left(r,\phi \right)+H_{z}^{scat}\left(r,\phi \right)\\ & =\underset{n\in Z}{\sum }i^{-n}J_{n}\left(k_{0}r\right)e^{in\left(\phi -\theta \right)}\\ & \;+\underset{n\in Z}{\sum }S_{n}H_{n}^{\left(2\right)}\left(k_{0}r\right)e^{in\left(\phi -\theta \right)}, \end{array}$$

where *J*_*n*_ and $H_{n}^{\left(2\right)}$ are the *n*th order Bessel function of the first kind and Hankel function of the second kind, respectively, *k*_0_ is the free space wavenumber, *θ* is the angle of the incident planewave, and *r* and *ϕ* are the polar coordinates [23]. The scattering coefficients *S*_*n*_ are obtained by applying the interface condition at *r* = *a* which is described by

(2) $$\left\{\begin{array}{c} H_{z,L}=H_{z,R},\;\\ \varepsilon_{L}^{-1}\mathrm{grad}\;H_{z,L}\cdot \hat{n}=\varepsilon_{R}^{-1}\mathrm{grad}\;H_{z,R}\cdot \hat{n},\; \end{array}\right.\;x\in \Gamma_{I},$$

where $\hat{n}$ is the interface normal and the *L*, *R* subscripts indicate the left and right elements of the interface. For perfect electric conductors, they are [23]

(3) $$S_{n}=-i^{-n}\frac{J_{n-1}\left(k_{0}a\right)-J_{n+1}\left(k_{0}a\right)}{H_{n-1}^{\left(2\right)}\left(k_{0}a\right)-H_{n+1}^{\left(n+2\right)}\left(k_{0}a\right)}.$$

Scattering coefficients for both gyrotropic and dielectric media can be described by the same expression [23]

(4) $$\begin{array}{cc} S_{n} & =-i^{-n}\frac{J_{n}\left(k_{0}a\right)}{H_{n}^{\left(2\right)}\left(k_{0}a\right)}C_{n},\\ C_{n} & =\frac{\frac{J_{n}^{\prime }\left(ka\right)}{J_{n}\left(ka\right)}-\left(\sqrt{\varepsilon_{\text{eff}}/\varepsilon_{0}}\frac{J_{n}^{\prime }\left(k_{0}a\right)}{J_{n}\left(k_{0}a\right)}-n\frac{\varepsilon_{g}}{ka\varepsilon_{t}}\right)}{\frac{J_{n}^{\prime }\left(ka\right)}{J_{n}\left(ka\right)}-\left(\sqrt{\varepsilon_{\text{eff}}/\varepsilon_{0}}\frac{H_{n}^{\prime }\left(k_{0}a\right)}{H_{n}\left(k_{0}a\right)}-n\frac{\varepsilon_{g}}{ka\varepsilon_{t}}\right)}. \end{array}$$

In the above, *ɛ*_0_ and $\varepsilon_{e\mathrm{ff}}=\varepsilon_{0}\frac{\varepsilon_{t}^{2}+\varepsilon_{g}^{2}}{\varepsilon_{t}}$ are the free-space and effective permittivity, respectively, where $\varepsilon_{g}=\frac{\omega_{p}^{2}\omega_{c}}{\omega \left(\omega_{c}^{2}-\omega^{2}\right)}$ and $\varepsilon_{t}=1+\frac{\omega_{p}^{2}}{\omega_{c}^{2}-\omega^{2}}$ are components of the permittivity tensor for a gyrotropic medium (Appendix A.1). Here, *ω* the field angular frequency, *ω*_*p*_ and *ω*_*c*_ the angular electron plasma and cyclotron frequencies, respectively [24]. Scattering coefficients for the reciprocal scenario are recovered when *ω*_*p*_ = 0.

The scattering coefficients *S*_*n*_ of order −1, 0, and 1 dominate the responses when the radius is much smaller than the background wavelength, i.e., *a* ≪ *λ*_0_. Then, we can approximate the scatterer by dipole moments, where the relation between their amplitude *d* = [*m*, *p*]^*T*^ and the incident field $F^{inc}={\left[H^{inc},\;E^{inc}\right]}^{T}$ is expressed as *d* = *αF* ^inc^, or

(5) $$\underset{=d}{\underset{︸}{\left[\begin{array}{c} m\\ p \end{array}\right]}}=\underset{=\alpha }{\underset{︸}{\left[\begin{array}{cc} \alpha^{00} & \alpha^{01}\\ \alpha^{10} & \alpha^{11} \end{array}\right]}}\underset{=F^{inc}}{\underset{︸}{\left[\begin{array}{c} H^{inc}\\ E^{inc} \end{array}\right]}}.$$

Here, *m* and *p* are magnetic and electric dipoles, respectively, and *α* is the polarizability tensor. Without loss of generality, due to the small size of the scatterers, no coupling between *H* and *E* is assumed, i.e., *α* ^01^ = *α* ^10^ = 0. Then, as shown in Appendix A.2, we have

(6) $$\begin{array}{cc} \alpha^{00} & =\frac{4}{k_{0}^{2}}iS_{0}\;\text{and}\\ \alpha^{11} & =\left[\begin{array}{cc} S_{1}-S_{-1} & -i\left(S_{1}+S_{-1}\right)\\ i\left(S_{1}+S_{-1}\right) & S_{1}-S_{-1} \end{array}\right]. \end{array}$$

In the above, we have *S*_−1_ = −*S*_1_ for a dielectric case, which implies reciprocity.

According to [25], the magnetic and electric field induced by a dipole can be expressed by *F* ^scat^ = Γ*d* or

(7) $$\underset{=F^{scat}}{\underset{︸}{\left[\begin{array}{c} H^{scat}\\ E^{scat} \end{array}\right]}}=\underset{=\Gamma }{\underset{︸}{\left[\begin{array}{cc} \Gamma_{00} & \Gamma_{01}\\ \Gamma_{10} & \Gamma_{11} \end{array}\right]}}\underset{=d}{\underset{︸}{\left[\begin{array}{c} m\\ p \end{array}\right]}},$$

where Γ is the dipole-dipole interaction matrix that maps a dipole to a scattered field (Appendix A.3).

The multi-scatterer generalization of eq. (5) for *i*th particle can be written as

(8) $$d_{i}=\alpha_{i}\underset{={\tilde{F}}_{i}^{inc}}{\underset{︸}{\left(F_{i}^{inc}+\overset{N}{\underset{j=1\;:\;i\neq j}{\sum }}\Gamma_{ij}d_{j}\right)}},\;i=1,2,\ldots ,N.$$

Here, the effective incident wave for the *i*th particle ${\tilde{F}}_{i}^{inc}$ is composed of the incident field $F_{i}^{inc}$ and scattered field induced by other particles Γ_*ij*_ *d*_*j*_, where Γ_*ij*_ is the dipole–dipole interaction matrix in eq. (7). *α*_*i*_ is the polarizability tensor for *i*th particle and the corresponding dipole moment is denoted by *d*_*i*_. Following the notation of a two-scatterer case in [26], (8) can be written as

(9) $$\underset{A^{-1}}{\underset{︸}{\left[\begin{array}{cccc} \alpha_{1}^{-1} & -\Gamma_{12} & \ldots & -\Gamma_{1N}\\ -\Gamma_{21} & \alpha_{2}^{-1} & \ldots & -\Gamma_{2N}\\ \vdots & \vdots & \ddots & \vdots \\ -\Gamma_{N1} & -\Gamma_{N2} & \ldots & \alpha_{N}^{-1} \end{array}\right]}}\underset{D}{\underset{︸}{\left[\begin{array}{c} d_{1}\\ d_{2}\\ \vdots \\ d_{N} \end{array}\right]}}=\underset{P}{\underset{︸}{\left[\begin{array}{c} F_{1}^{inc}\\ F_{2}^{inc}\\ \vdots \\ F_{N}^{inc} \end{array}\right]}}.$$

Here, each element of *D* is the dipole moment *d* = [*m p*]^*T*^ induced by each particle. Each component of the vector *P* corresponds to the incident field *F* ^inc^ sampled at the position of each particle:

(10) $$P=\underset{C}{\underset{︸}{\left[\begin{array}{c} \int d\Omega \delta \left(x-x_{1}\right)\\ \int d\Omega \delta \left(x-x_{1}\right)\\ \vdots \\ \int d\Omega \delta \left(x-x_{n}\right) \end{array}\right]}}F^{inc},$$

where Ω is the domain that contains all particles and *δ*(*x* − *x*_*i*_) is a Dirac delta.

Similarly, the generalization of (7) for an arbitrary position *x* with an *N*-particle system is

(11) $$F^{scat}=\underset{G}{\underset{︸}{\left[\begin{array}{cccc} \Gamma_{xx_{1}} & \Gamma_{xx_{2}} & \ldots & \Gamma_{xx_{N}} \end{array}\right]}}D,$$

where each interaction matrix $\Gamma_{xx_{i}}$ relates the field at *x* due to the dipole at *x*_*i*_. Rearranging and substituting (9) into *D* of (11), then replacing *P* with (10), the relationship between the scattered field to the incoming field can be obtained as

(12) $$F^{scat}={GACF}^{inc}.$$

The incident field can be expanded in a functional basis of our choice [5], [12], [25], that is, expressed as a summation of basis functions with distinct coefficients. These coefficients serve as control variables encoding the input signal [5]. Then, the complexity of the problem is governed by the number of input and output ports. Let *B*_*n*_(*x*) = *J*_*n*_(*k*_0_ *r*)*e* ^in(*ϕ*−*θ*)^ represents the basis for the incident magnetic field *H* ^inc^, or the input ports. Then, the expansion reads

(13) $$F^{inc}=\underset{n}{\sum }c_{n}^{in}g_{n}\left(x\right),$$

where $g_{n}\left(x\right)={\left[B_{n}\left(x\right),\;{\left(i\omega \varepsilon \right)}^{-1}\mathrm{curl}\;B_{n}\left(x\right)\right]}^{T}$ . The coefficient $c_{n}^{in}$ determines the amplitude of each port, and can be varied to represent the desired input function.

Likewise, the choice of output ports is motivated by the general solution of the scattered field in an exterior problem, i.e.,

(14) $$H^{scat}=\underset{m}{\sum }c_{m}^{out}h_{m}\left(x\right),$$

where $h_{m}\left(x\right)=H_{m}^{\left(2\right)}\left(k_{0}r\right)e^{im\left(\phi -\theta \right)}$ . Let *h* ^*m*^(*x*) denotes the dual function of *h*_*m*_(*x*). Then, the output coefficients $c_{m}^{out}$ of (14) are obtained by

(15) $$c_{m}^{out}=\int d\Omega \left(h^{m}\left(x\right)H^{scat}\right).$$

It is noted that the choice of basis functions is, in general, arbitrary; the above selection ensures completeness for the two-dimensional problem [27].

Let $Q={\left[1,\;0\right]}^{T}$ denotes a linear operator that extracts magnetic field *H* from the field vector *F* such that *H* ^scat^ = *QF* ^scat^. Then, eq. (12) can be expressed in terms of input and output ports as the scattering matrix equation:

(16) $$c_{m}^{out}=S_{mn}c_{n}^{in}=\int d\Omega \left(h^{m}QGACg_{n}\right)c_{n}^{in},$$

or in tensor form,

(17) $$c^{out}=Sc^{in}.$$

The scattering matrix **S**_**mn**_ is a linear operator mapping the input vector **c** ^**in**^ to the output vector **c** ^**out**^.

### 2.2 Mathematical problem

The target linear mathematical problem for any ODE can be stated as

(18) A[u]=f,

where *A* is the operator acting on an unknown function *u* and *f* is a given function. The operator *A* is not necessarily self-adjoint, which may describe a nonreciprocal physical problem. For example, we consider the following operator, which represents a typical form of a second-order boundary value problem:

(19) $$A\left[\cdot \right]=\frac{d}{dx}\left[\alpha \frac{d}{dx}\cdot \right]+\beta ,$$

with two complex-valued parameters *α* and *β* and the periodic boundary condition over 2*π*. In the weak form, the above strong form can be expressed as

(20) $$\int_{0}^{2\pi }\left[\frac{dv}{dx}\alpha \frac{du}{dx}-v\beta u\right]dx=\int_{0}^{2\pi }vfdx,\;\forall v\in V.$$

In the above, *v* denotes a test function, and *V* is a set of admissible functions.

Among many choices of finite-dimensional approximations, we take Rayleigh-Ritz type discretization, which reads

(21) $$\begin{array}{cc} v\left(x\right) & =\overset{N}{\underset{m=-N}{\sum }}v_{m}g_{m}\left(r\right)=\overset{N}{\underset{m=-N}{\sum }}v_{m}e^{-imr},\\ u\left(x\right) & =\overset{N}{\underset{n=-N}{\sum }}u_{n}h_{n}\left(r\right)=\overset{N}{\underset{n=-N}{\sum }}u_{n}e^{\mathrm{inr}}. \end{array}$$

The expansion is evaluated at the interface with a radius of choice *r* = *a*. The above basis choice implies that we use sinusoidal basis for both input and output ports in (13) and (15). Thus, *f* is now expressed with a finite series of sinusoidal functions representing the input.

Using this result, eq. (18) can be approximated to a matrix equation

(22) Au=f,

where **u** and **f** are the vectors of the coefficients *u*_*n*_, *f*_*n*_ defined in eq. (21), respectively. The operator ***A*** is the finite approximation of the operator $A\left[\cdot \right]$ , whose elements are calculated using

(23) $$A_{mn}=\frac{1}{2\pi }\int_{0}^{2\pi }\left(-mn\alpha -\beta \right)e^{i\left(m+n\right)x}dx.$$

The aim is to design a scatterer such that its geometry maps the response of eq. (22) to its scattering matrix in a way that the solution is imprinted on the scattering fields when illuminated by incident fields **f**. Therefore, the scattering matrix **S** should satisfy

(24) SA=AS=γI,

where *I* is the identity matrix and *γ* is a scaling factor [5].

### 2.3 Scatterer design

The position of the particles can be formed as an inverse design problem. From eq. (24), it follows that the cost function for this inverse design problem can be formulated as

(25) $$C\left(\gamma ,p\right)=\frac{1}{2}\overset{N}{\underset{j=-N}{\sum }}\|\gamma^{-1}SA_{j}-I{\|}_{2}^{2},$$

where $\|\ldots {\|}_{2}^{2}$ is the square of the Euclidean norm and *p* is the position vector of the particles. The scaling factor is optimized in addition to the positions to introduce an extra degree of freedom in the optimization problem. A new vector can be formed *q* = (*γ*, *p*), which is the optimization variable. A constant number of *D*_*c*_ dielectric and *M*_*c*_ gyrotropic cylinders were chosen at the start of each optimization, the optimization variable is defined

(26) $$\begin{array}{c} q={\left[\gamma^{-1},p_{x}^{1},p_{y}^{1},\ldots ,p_{x}^{D_{c}+M_{c}},p_{y}^{D_{c}+M_{c}}\right]}^{T}\\ \text{subject}\;\text{to}:-R/2\leq q_{j}^{k}\leq R/2,\;\forall k\geq 2,\\ 0.5\leq \gamma^{-1}\leq 3, \end{array}$$

where $p_{j}^{i}$ is the position of the *i*th particle in the *j* axis and *R* is a positional constraint. This constraint limits the optimization region to a square region of size *R* × *R* to ensure that: (a) the scatterers remain within the simulation limits and, (b) the desired compactness is achieved. Since the multiplier is an unbounded variable, the minimum constraint on *γ* ^−1^ was enforced to avoid a singularity in eq. (24), while the maximum value was empirically chosen.

**Figure 2:**
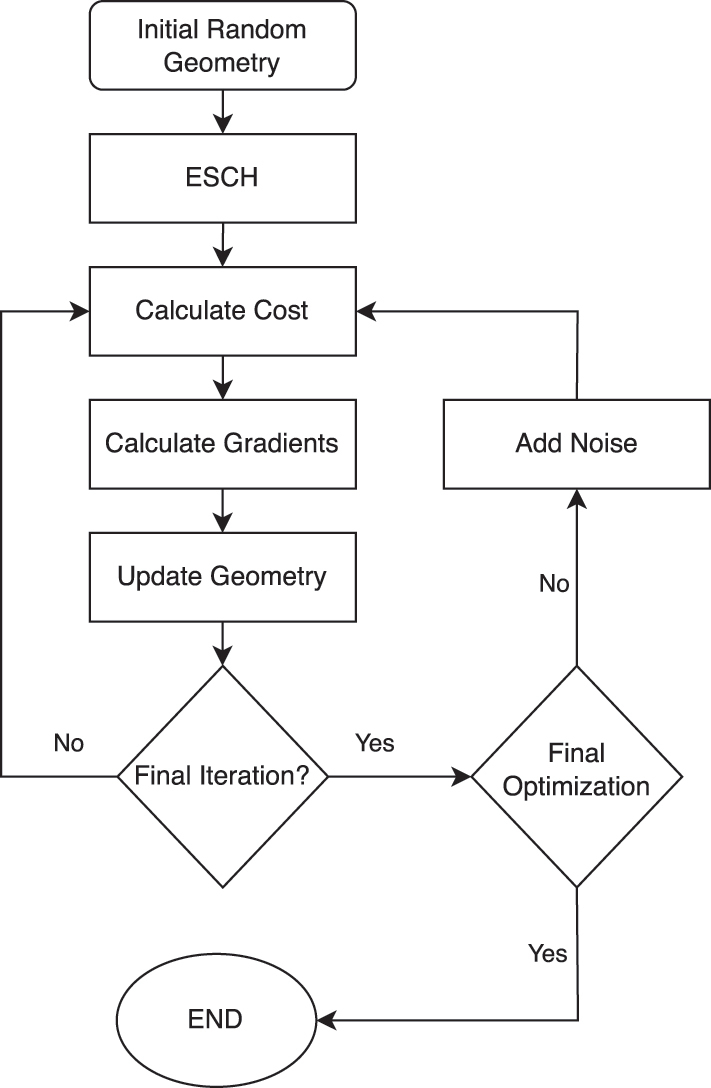
Optimization algorithm for the positions of the cylinders.

A two-step optimization process is used as shown in Figure 2 and the code can be found in [28]. The framework was implemented using the open source library NLopt in Python [29]. First, an optimization is performed using the global evolutionary algorithm (ESCH) [30] followed by the gradient based method of moving asymptotes (MMA) [31]. As a global optimizer is better suited to exploring a larger part of the parameter space, introducing it at the beginning allows for better exploration of a wider region of possible solutions. However, as global optimizers often suffer from slow convergence, the gradient based optimizer allows for faster convergence to the local minima of the region that the ESCH algorithm identified. The hybrid method explores the solution space more efficiently and accurately than deploying either of the algorithms on an individual basis [32].

The calculation of gradients *δq*, required by MMA, was performed using finite differences. Given the small number of elements in vector *q*, the problem did not necessitate the use of the adjoint variable method. To reduce the computational time required, the process was parallelized using mpi4py [33] to calculate each element of *δq* on a different simultaneous process.

Random noise was integrated in the optimization process to encourage tolerance in the solution, yielding more robust devices. The gradient based optimizer was employed 10 times with a maximum number of iterations at 1,000 per optimization session. Each time the position vector with the lowest cost function was used with added noise i.e.

(27) $$\begin{array}{cc} q_{j} & =q_{best}+Y\times \lambda_{0}\times 10^{-3},\\ & \;Y_{k}\in \left[0,1\right),0\leq j\leq 2\left(D_{c}+M_{c}\right), \end{array}$$

where *q*_best_ is the best performing geometry from the previous iterations and *Y* is a random variable vector and *Y*_*k*_ is the *k*th element of *Y*. This can also be considered a fabrication robustness measure, with geometries who are less susceptible to this perturbation being favored as the starting point for the next optimization.

## 3 Results

**Figure 3:**
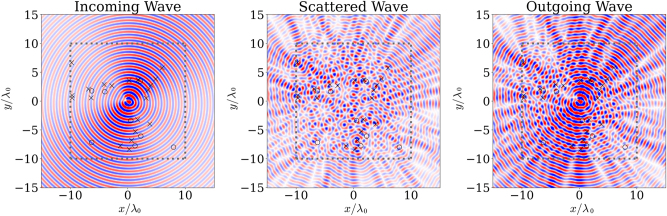
Plot showing the incoming, scattered and outgoing *H*_*z*_ from an analog computing scatterer. The dashed line shows the optimization region, ‘*x*’ represents gyrotropic scatterers, while ‘*o*’ represents dielectric scatterers.

Using the quasi-static dipolar resonant condition from [23], the design frequency of *ω* = 1 × 10^12^ rad s^−1^ was chosen with *ω*_*p*_ = 2.1 × 10^12^ rad s^−1^ and *ω*_*c*_ = 0.57*ω*_*p*_ for gyrotropic cylinders, and loss-less silicon as the dielectric material with *ɛ* = 12*ɛ*_0_, *ω*_*c*_ = 0 for a 7 harmonics system. The optimization region is a 20*λ*_0_ × 20*λ*_0_ square as shown in Figure 3. The figure also shows how the scatterer interacts with the 7 incoming spherical harmonics to map the solution to the scattered fields.

The scatterer in Figure 3 shows the incident and scattered fields to a scatterer constructed with a $\alpha \left(x\right)=\sum_{n=-3}^{3}a_{n}e^{\mathrm{inx}}$ and $\beta \left(x\right)=\sum_{n=-3}^{3}b_{n}e^{\mathrm{inx}}$ , where

(28) $$\left[a_{n}\right]=\frac{1}{4\pi^{2}}\left[\begin{array}{c} \frac{\left(1+2i\right)}{\sqrt{2}}\\ 0.75+0.35i\\ 1\\ 2.25-1i\\ 4.5+1i\\ -6+3.5i\\ 1.5-2.75i \end{array}\right],\;\left[b_{n}\right]=\frac{1}{4\pi^{2}}\left[\begin{array}{c} 6.5+1.75i\\ 11-11i\\ 4+1.75i\\ 59+79i\\ 2+1i\\ 14+6i\\ -1.5+0.1i \end{array}\right].$$

**Figure 4:**
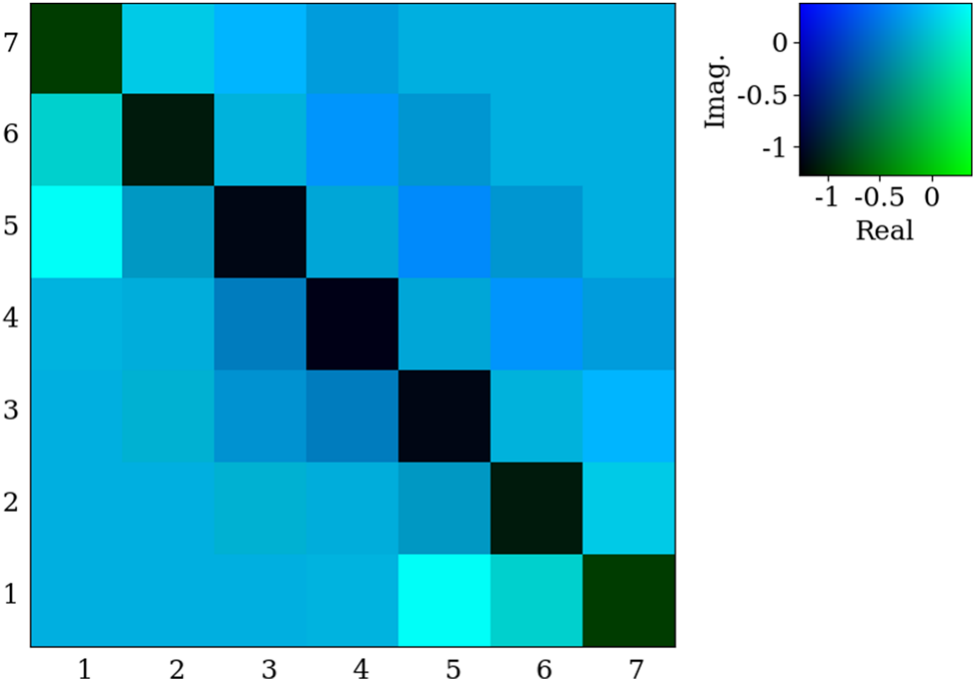
Example inverse non-Hermitian kernel used in the cost function to map the scattering matrix on the scatterer’s geometry using eq. (25).

**Figure 5:**
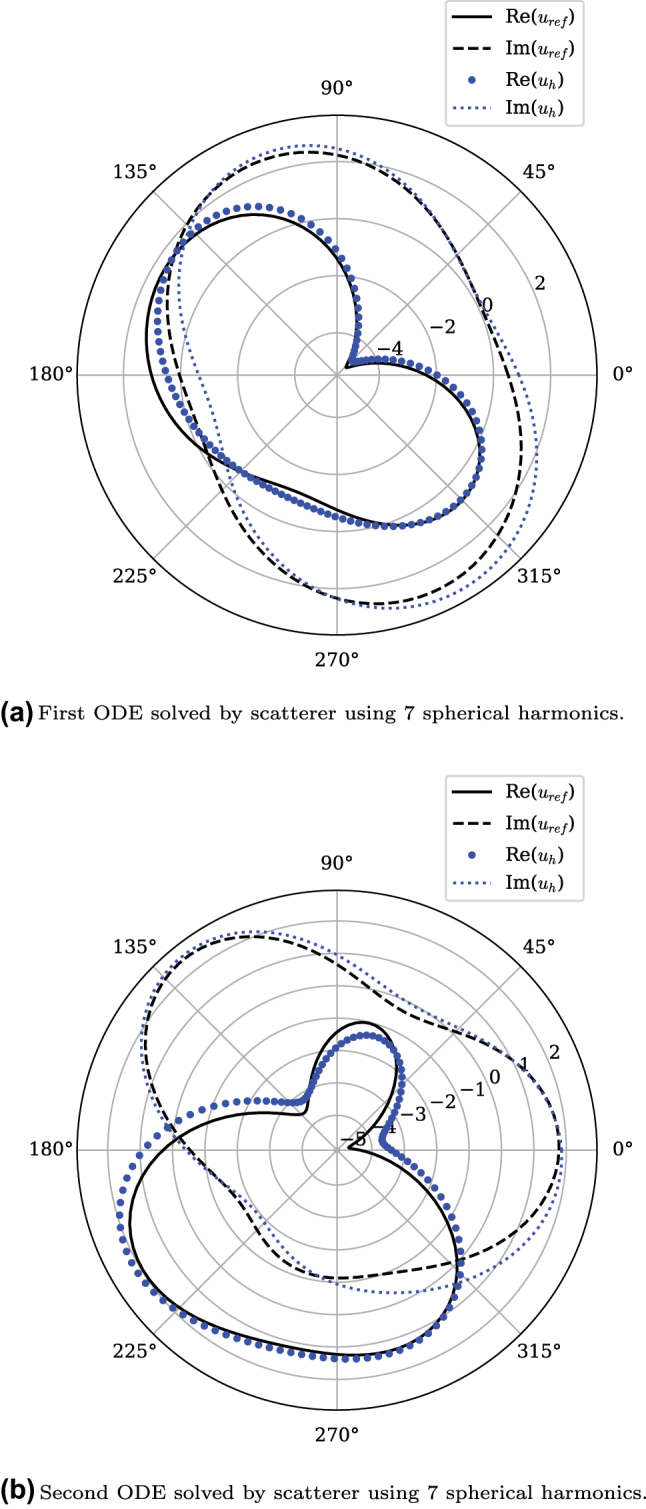
Solution to two different ODEs by the same scatterer shown in Figure 3. (a) First ODE solved by scatterer using 7 spherical harmonics. (b) Second ODE solved by scatterer using 7 spherical harmonics.

The performance of the scatterer was tested using two input functions *f*_*a*,*b*_.

(29) $$f_{a}\left(x\right)=\left\{\begin{array}{c} \frac{\left(1+1i\right)}{\sqrt{2}},\\ 2,\\ 3.5,\\ 1.0,\\ 3,\\ -2,\\ \frac{\left(1-1i\right)}{\sqrt{2}}, \end{array}\right.\;f_{b}\left(x\right)=\left\{\begin{array}{c} \left(1+1i\right)/\sqrt{2},\\ \left(1-1i\right)/\sqrt{2},\\ 2.0i,\\ \left(2+1i\right)/\sqrt{2},\\ \left(2-1i\right)/\sqrt{2},\\ \left(1+2i\right)/\sqrt{2},\\ \left(1i\right)/\sqrt{2}, \end{array}\right.$$

where each element represents the value at $\frac{2\pi }{7}$ intervals of *x* with 0 ≤ *x* ≤ 2*π*. *f*_*a*_ and *f*_*b*_ were used for the inputs in Figure 5a and b, respectively. The 7 by 7 target kernel A was used to find the scattering matrix, shown in Figure 4 was constructed with *α* and *β*. The non-Hermitian matrix shows the inverse of the target in a real-imaginary color map. The symmetry of the matrix is visibly broken along the main diagonal.

Figure 5 shows two solutions of ODEs from the same scatterer, the design of the scatterer is shown in Figure 3. In both cases the solution of the analog solver closely matches the numerical solution. Both problems have increased complexity compared to previously demonstrated analog computing scatterers [5]. Compared to numerical solutions, a 5 by 5 scatterer achieved a maximum error of ∼1 %, while for the 7 by 7 scatterer presented here, the maximum error was ∼3 %. While the accuracy of numerical solutions, such as *u*_ref_, depends on a range of factors, it is generally accepted that they can achieve accuracies ≪1 % [34]. The authors believe, however, that the permitted trade-off between accuracy and efficiency, as well as their compactness make this approach attractive for implementing future analog computing devices.

**Figure 6:**
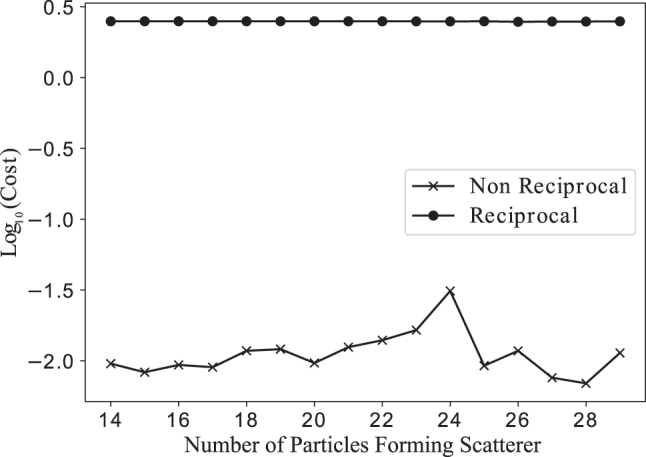
The logarithm of the smallest achieved cost function of eq. (25) when optimizing with gyrotropic media (non-reciprocal) or perfect electric conductors (reciprocal).

To understand whether gyrotropic media are essential for the solution of generally asymmetric ODEs, their performance was compared to scatterer geometries with an ensemble of perfect electric conductor (PEC) and dielectric cylinders. Since gyrotropic media are PEC with an external magnetic bias, the initial conditions of the previous experiment are repeated, without the external magnetic bias. Figure 6 shows the performance of the best design for each case for different numbers of scatterer particles. The use of non-reciprocal media clearly results in improved performance for the solution of non-reciprocal ODEs. Using gyrotropic cylinders consistently gives a lower cost function by more than an order of magnitude (or 95 % reduction). It is noted that in both cases there is slight fluctuation, believed to be due to: (a) random starting conditions, (b) increasing the number of optimization parameters, and (c) the ability of a larger number of particles to populate larger matrices. Using the literature value for the lossy permittivity of high resistivity silicon at *ω* = 1 THz, *ɛ* = 11.66 + 2.4*i* × 10^−3^ [35] results in a difference <1 % in the cost function for all numbers of particles, demonstrating the robustness of the solution.

**Figure 7:**
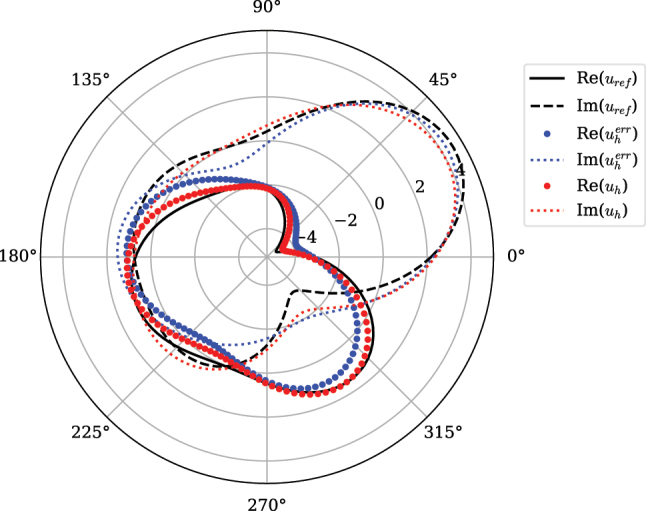
Comparison of the performance of the scatterer with the ideal solution (black), unperturbed scatterer (red) and uniformly perturbed scatterer (blue).

To test the robustness of the system, the optimal solution was perturbed using eq. (27) with *Y* again generated using a uniform random distribution with boundaries $\left[0,1\right)$ . Figure 7 shows that effect, with the perturbed solution $u_{h}^{err}$ closely following the unperturbed and reference solutions with the perturbed system’s relative error at 8 % for the real part and 9 % for the imaginary part compared to the numerical solution. For the test, a third input function *f*_*c*_ was trialed

(30) $$f_{c}\left(x\right)=\left\{\begin{array}{ccc} \left(1+1i\right)/\sqrt{2}, & \; & 0<x\leq 2\pi /7,\\ \left(1+1i\right)/\sqrt{2}, & \; & 2\pi /7<x\leq 4\pi /7,\\ 2.0i & , & 4\pi /7<x\leq 6\pi /7,\\ \left(2+1i\right)/\sqrt{2}, & \; & 6\pi /7<x\leq 8\pi /7,\\ \left(2-1i\right)/\sqrt{2}, & \; & 8\pi /7<x\leq 10\pi /7,\\ \left(1-2i\right)/\sqrt{2}, & \; & 10\pi /7<x\leq 12\pi /7,\\ 1/\sqrt{2}, & \; & 12\pi /7<x<2\pi . \end{array}\right.$$

The third example highlights not only the small deviation between perturbed scatterers’s solution to the unperturbed but also to the analytical solution. This robustness to errors in position is an important step towards a practical implementation of the analog computing device. This can be attributed to embedding noise in the optimization problem, biasing the final result to geometries that have small variations in solution under such errors.

## 4 Conclusions

In this paper, an inverse-design technique was used to create a system of gyrotropic and dielectric scatterers whose electromagnetic scattering characteristics can be used to implement an analog computing device capable of solving ODEs. The input function for the scatterer is encoded in the spherical harmonics of an incident electromagnetic wave that is scattered from the system. The solution can then be read from the spherical harmonics of the scattered fields. The optimal geometries were found using a combination of global and local optimizers to determine the position of the gyrotropic and dielectric cylinder scatterers. As a proof of concept, scatterers using 7 spherical harmonics were designed and tested against two different ODEs, and a reasonable level of accuracy was demonstrated.

Similar analog computing scatterers using PEC-only scatterers were optimized using the same method in order to understand the necessity of gyrotropic media. The PEC-based scatterers achieved cost functions higher than the gyrotropic based solutions by an order of magnitude. While the gyrotropic based scatterers showed an improved performance as the number of optimized parameters was increased, the PEC-based scatterers maintained the same level of performance.

Expanding on the ideas introduced by previous scattering-based analog computing devices [5], we used inverse design to develop non-reciprocal scattering based analog computers. We believe that these ultra-fast and compact devices can be deployed for problem-specific non-reciprocal applications.

## A Appendix

### A.1 Permittivity and permeability for a gyrotropic medium

The permittivity and the permeability for a (piecewise) homogeneous gyrotropic medium reads [23]

(31)
$$\varepsilon =\varepsilon_{o}\left[\begin{array}{ccc} \varepsilon_{t} & i\varepsilon_{g} & 0\\ -i\varepsilon_{g} & \varepsilon_{t} & 0\\ 0 & 0 & \varepsilon_{a} \end{array}\right]\;\text{and}\;\mu =\mu_{o}.$$

In the above,

(32)
$$\varepsilon_{t}=1+\frac{\omega_{p}^{2}}{\omega_{c}^{2}-\omega^{2}}\;\text{and}\;\varepsilon_{g}=\frac{\omega_{p}^{2}\omega_{c}}{\omega \left(\omega_{c}^{2}-\omega^{2}\right)}.$$

We assume a transverse-electric incident plane wave with no external current density, i.e.,

(33)
$$H_{x}=H_{y}=0,\;\frac{\partial H}{\partial z}=0,\;\text{and}\;J=0.$$

Then, the Maxwell’s equations are reduced to a two-dimensional Helmholtz equation in the *z* component of the vector field *H*, i.e.,

(34)
$$div\;\varepsilon_{e\mathrm{ff}}^{-1}\mathrm{grad}\;H_{z}+\omega^{2}\mu H_{z}=0,$$

where the effective permittivity reads

(35)
$$\varepsilon_{e\mathrm{ff}}=\varepsilon_{o}\frac{\varepsilon_{t}^{2}-\varepsilon_{g}^{2}}{\varepsilon_{t}}.$$

### A.2 Derivation of *α* ^00^ and *α* ^11^

Electric and magnetic fields due to an electric dipole moment located at *ρ*′ = (*r*′, *ϕ*′) are [25]

(36)
$$E=\varepsilon_{0}^{-1}\left[\left(p\cdot \mathrm{grad}\right)\mathrm{grad}\;g+k_{0}^{2}pg\right]\;\text{and}$$

(37)
H=−iωp×gradg.

In the above, we have

(38)
$$g\left(\rho ;\rho^{\prime }\right)=\frac{i}{4}H_{0}^{\left(2\right)}\left(k_{0}\left|\rho -\rho^{\prime }\right|\right).$$

Let *ρ*′ = 0 and considering only the electric dipole contribution of the scattered field, i.e. *n* = −1, 1, the left-hand-side of the (37) becomes

(39)
$$H_{z}^{\text{scat}}=\underset{n\in Z}{\sum }S_{n}H_{n}^{\left(2\right)}\left(k_{0}r\right)e^{in\left(\phi -\theta \right)}$$

(40)
$$\begin{array}{ll} & \approx S_{-1}H_{-1}^{\left(2\right)}\left(k_{0}r\right)e^{-i\left(\phi -\theta \right)}+S_{1}H_{1}^{\left(2\right)}\left(k_{0}r\right)e^{i\left(\phi -\theta \right)}\\ & =\left[\left(S_{1}e^{-i\theta }-S_{-1}e^{i\theta }\right)\cos\phi +i\left(S_{1}e^{-i\theta }\right.\right.\\ & \;\left.\left.+S_{-1}e^{i\theta }\right)\sin\phi \right]H_{1}^{\left(2\right)}\left(k_{0}r\right). \end{array}$$

Note that we have *S*_−1_ = −*S*_1_ for reciprocal cases. The right-hand-side of (37) becomes

(41)
$$\begin{array}{ll} -i\omega p\times \mathrm{grad}\;g & =-i\omega \left(p_{x}\frac{\partial g}{\partial y}-p_{y}\frac{\partial g}{\partial x}\right)\\ & =-\frac{\omega k_{0}}{4}\frac{H_{1}^{\left(2\right)}\left(k_{0}r\right)}{r}\left(p_{x}y-p_{y}x\right)\\ & =-\frac{\omega k_{0}}{4}H_{1}^{\left(2\right)}\left(k_{0}r\right)\left(p_{x}\sin\phi -p_{y}\cos\phi \right). \end{array}$$

Then, we have

(42)
$$\begin{array}{ll} p_{x} & =-\frac{4i\left(S_{1}e^{-i\theta }+S_{-1}e^{i\theta }\right)}{\omega k_{0}}\;\text{and}\\ p_{y} & =\frac{4\left(S_{1}e^{-i\theta }-S_{-1}e^{i\theta }\right)}{\omega k_{0}}. \end{array}$$

On the other hand, the polarizability is defined as

(43)
$$p=\alpha^{11}E^{inc},$$

where

(44)
$$\begin{array}{ll} \varepsilon_{0}\left(i\omega \right)E^{inc} & ={\left.H^{inc}\right|}_{\rho^{\prime }=0}\\ & ={\left.e^{-ik_{0}x\cos\theta }e^{-ik_{0}y\sin\theta }{\hat{e}}_{z}\right|}_{\rho^{\prime }=0}\\ & =-ik_{0}\sin\theta {\hat{e}}_{x}+ik_{0}\cos\theta {\hat{e}}_{y}\\ & =\frac{k_{0}}{2}\left({\text{e}}^{-\text{i}\theta }-{\text{e}}^{\text{i}\theta }\right){\hat{e}}_{x}+\frac{ik_{0}}{2}\left({\text{e}}^{-\text{i}\theta }+{\text{e}}^{\text{i}\theta }\right){\hat{e}}_{y}. \end{array}$$

Combining (42), (43), and (44), we have a system of linear equations, i.e.,

(45)
$$\left\{\begin{array}{c} -\frac{4iS_{1}}{\omega k_{0}}=\frac{k_{0}}{2\varepsilon_{0}\left(i\omega \right)}\left[\alpha_{xx}+i\alpha_{xy}\right],\;\\ -\frac{4iS_{-1}}{\omega k_{0}}=\frac{k_{0}}{2\varepsilon_{0}\left(i\omega \right)}\left[-\alpha_{xx}+i\alpha_{xy}\right],\;\\ \frac{4S_{1}}{\omega k_{0}}=\frac{k_{0}}{2\varepsilon_{0}\left(i\omega \right)}\left[\alpha_{yx}+i\alpha_{yy}\right],\;\\ -\frac{4S_{-1}}{\omega k_{0}}=\frac{k_{0}}{2\varepsilon_{0}\left(i\omega \right)}\left[-\alpha_{yx}+i\alpha_{yy}\right],\; \end{array}\right.$$

or

(46)
$$\frac{k_{0}}{2\varepsilon_{0}\left(i\omega \right)}\left[\begin{array}{cccc} 1 & i & 0 & 0\\ -1 & i & 0 & 0\\ 0 & 0 & 1 & i\\ 0 & 0 & -1 & i \end{array}\right]\left(\begin{array}{c} \alpha_{xx}\\ \alpha_{xy}\\ \alpha_{yx}\\ \alpha_{yy} \end{array}\right)=\frac{4i}{\omega k_{0}}\left(\begin{array}{c} -S_{1}\\ -S_{-1}\\ -iS_{1}\\ iS_{-1} \end{array}\right).$$

The solution to the above system of equations is

(47)
$$\alpha^{11}=\left[\begin{array}{cc} \alpha_{xx} & \alpha_{xy}\\ \alpha_{yx} & \alpha_{yy} \end{array}\right]=\frac{4\varepsilon_{0}}{k_{0}^{2}}\left[\begin{array}{cc} S_{1}-S_{-1} & -i\left(S_{1}+S_{-1}\right)\\ i\left(S_{1}+S_{-1}\right) & S_{1}-S_{-1} \end{array}\right].$$

Next, we derive *α* ^00^ such that

(48)
$$m=\alpha^{00}H^{inc},$$

where [25]

(49)
$$H^{\text{scat}}=-mk^{2}g.$$

We assume that *S*_0_, *S*_−1_, and *S*_1_ dominate the response in (39). The contributions of *S*_−1_ and *S*_1_ have been addressed previously. Thus, considering only the remaining *S*_0_ term, we obtain

(50)
$$\alpha^{00}=\frac{4iS_{0}}{k_{0}^{2}}.$$

### A.3 Elements of Γ

Expansion of the elements in eq. (7)

(51)
$$\begin{array}{cc} \Gamma_{00} & =\frac{k_{0}^{2}i}{4}H^{\left(0\right)}\left(k_{0}r\right),\\ \Gamma_{01} & =-\frac{ck_{0}^{3}}{4}\frac{\left(y_{i}-y_{j}\right)}{r}H^{\left(1\right)}\left(k_{0}r\right),\\ \Gamma_{10} & =\frac{k_{0}^{3}}{4\varepsilon_{0}c}\frac{\left(y_{i}-y_{j}\right)}{r}H^{\left(1\right)}\left(k_{0}r\right),\\ \Gamma_{11} & =\frac{k_{0}^{4}}{4\varepsilon_{0}}\left(\frac{{\left(y_{i}-y_{j}\right)}^{2}}{r^{2}}H^{\left(0\right)}\left(k_{0}r\right)\right.\\ & \;\left.+\frac{{\left(x_{i}-x_{j}\right)}^{2}-{\left(y_{i}-y_{j}\right)}^{2}}{k_{0}r^{3}}H^{\left(1\right)}\left(k_{0}r\right)\right). \end{array}$$
